# Supplementary material for: Proteome-wide prediction of the mode of inheritance and molecular mechanisms underlying genetic diseases using structural interactomics
Source: iScience. 2025 Jun 4;28(7):112812. doi: 10.1016/j.isci.2025.112812 (PMC12209950; doi:10.1016/j.isci.2025.112812)
Supplement: Document S1. Figures S1–S8 [file mmc1.pdf]

iScience, Volume *28*

Supplemental information

Proteome-wide prediction of the mode of inheritance and molecular mechanisms underlying genetic diseases using structural interactomics

Ali Saadat and Jacques Fellay

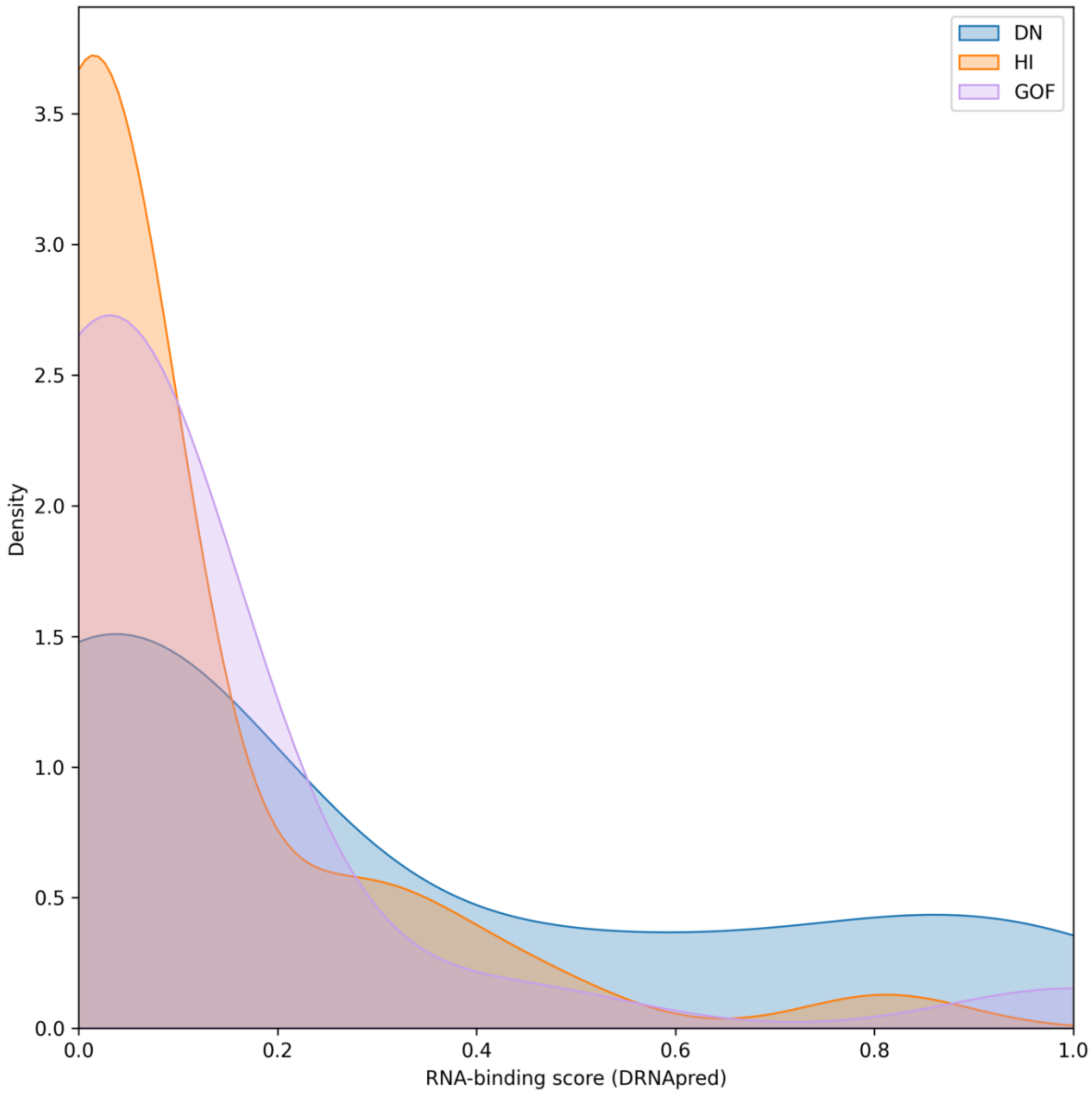

Figure S1: Distribution of RNA-binding scores based on DRNApred.

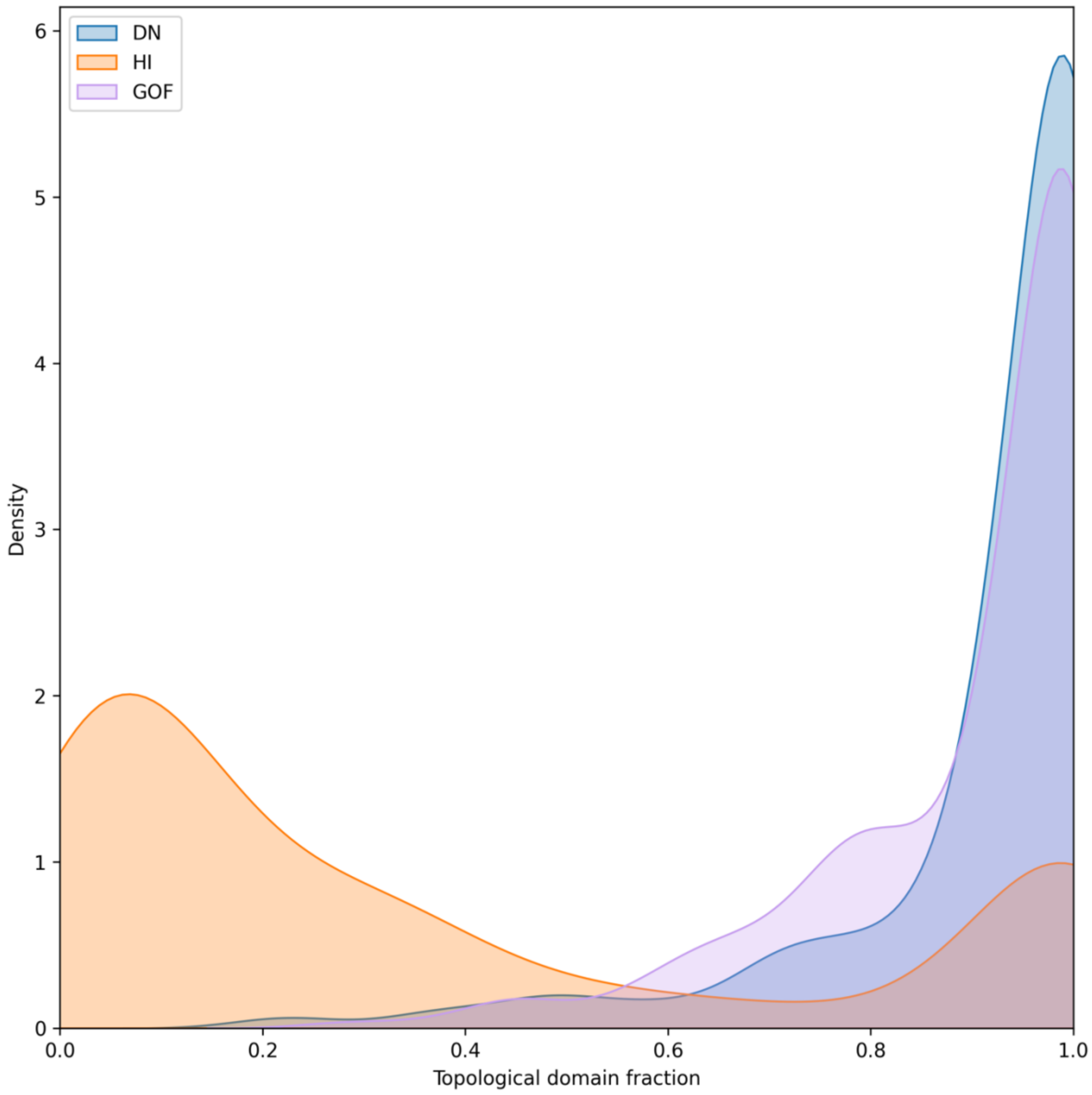

Figure S2: The distribution of the fraction of topological domain based on UniProt annotations.

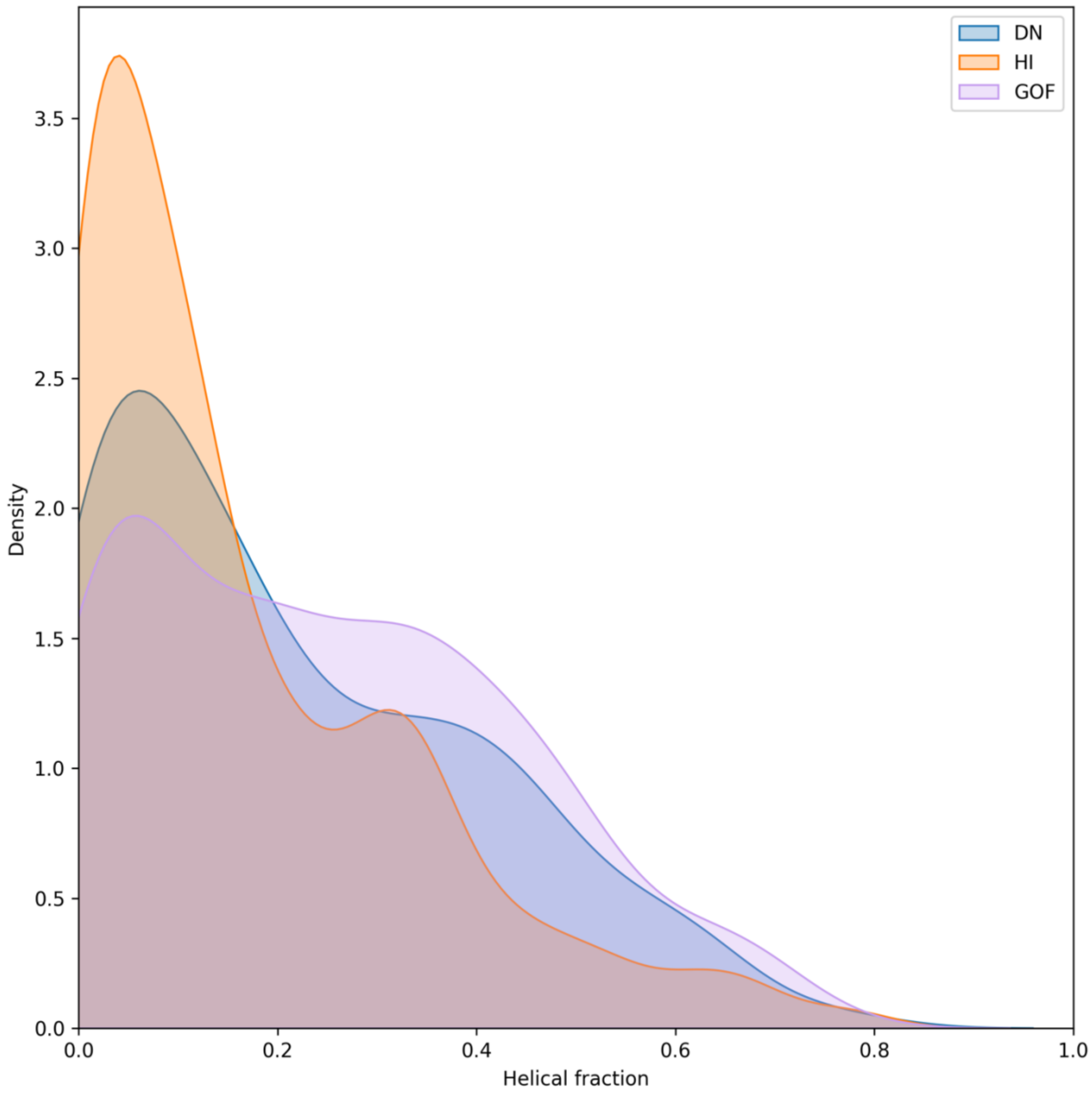

Figure S3: The distribution of the fraction of helical residues based on UniProt annotations.

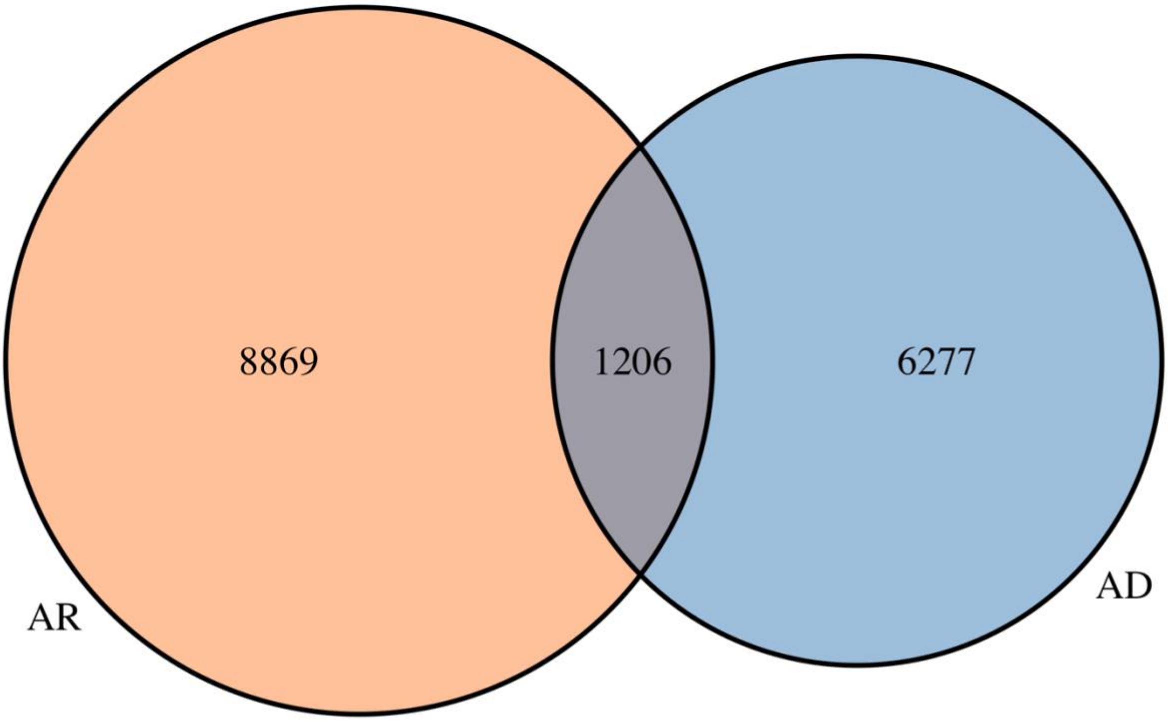

Figure S4: Number of predicted AD, AR, and ADAR based on the selected GAT model.

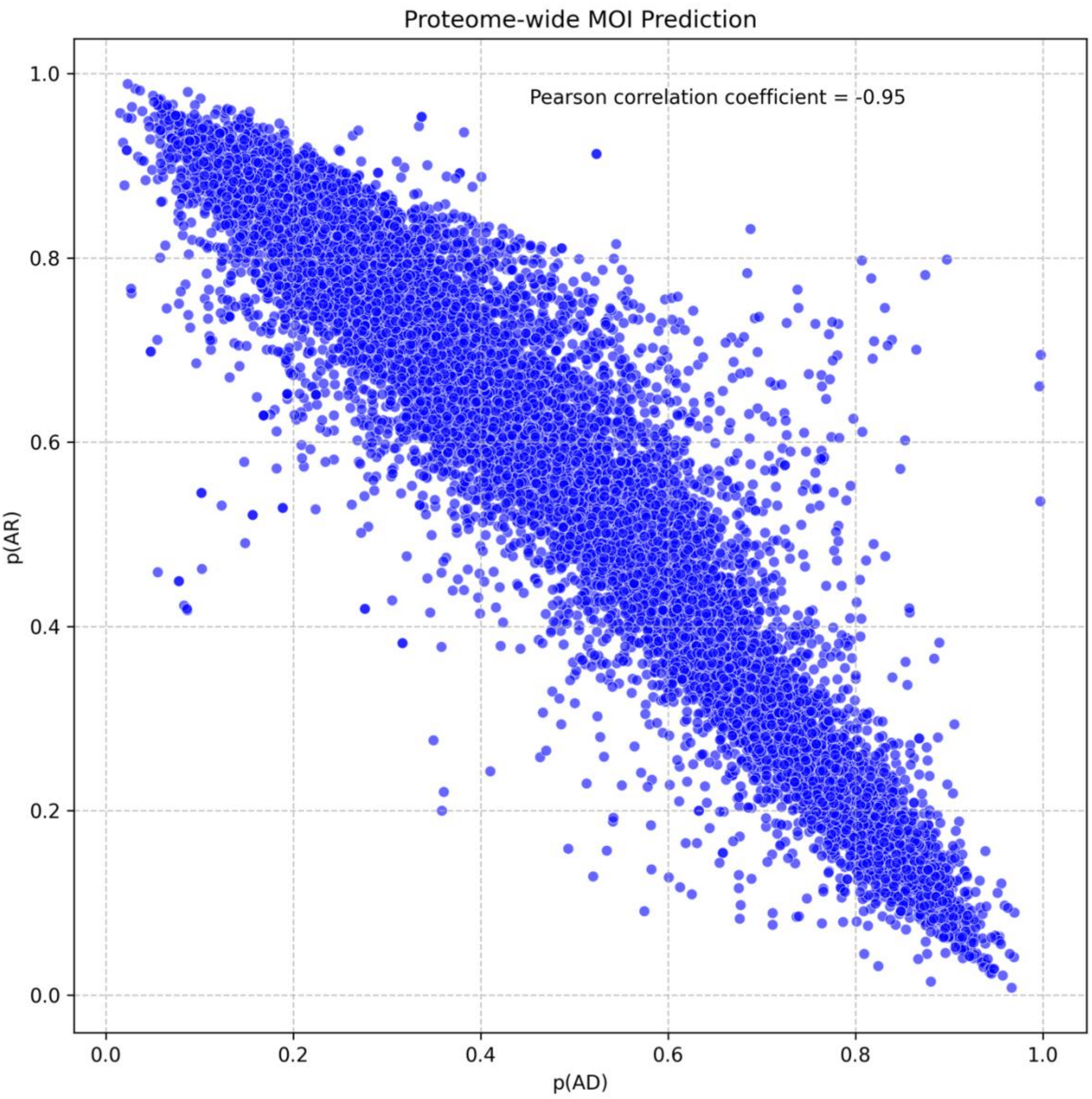

Figure S5: probability of AD (pAD) vs probability of AR (pAR) for all autosomal proteins.

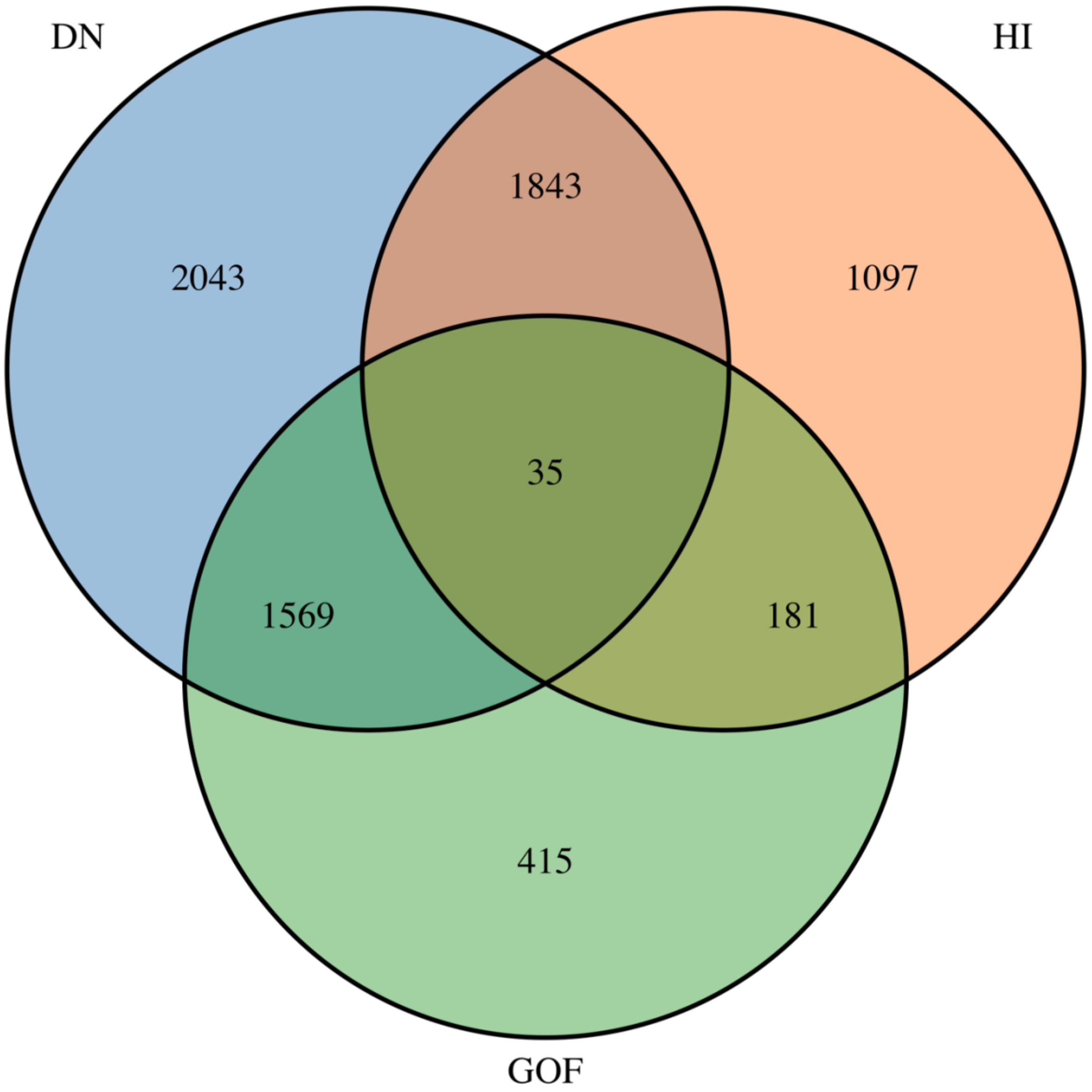

Figure S6: Number of proteins predicted based on the selected GCN model. Prediction was performed on all AD and ADAR proteins.

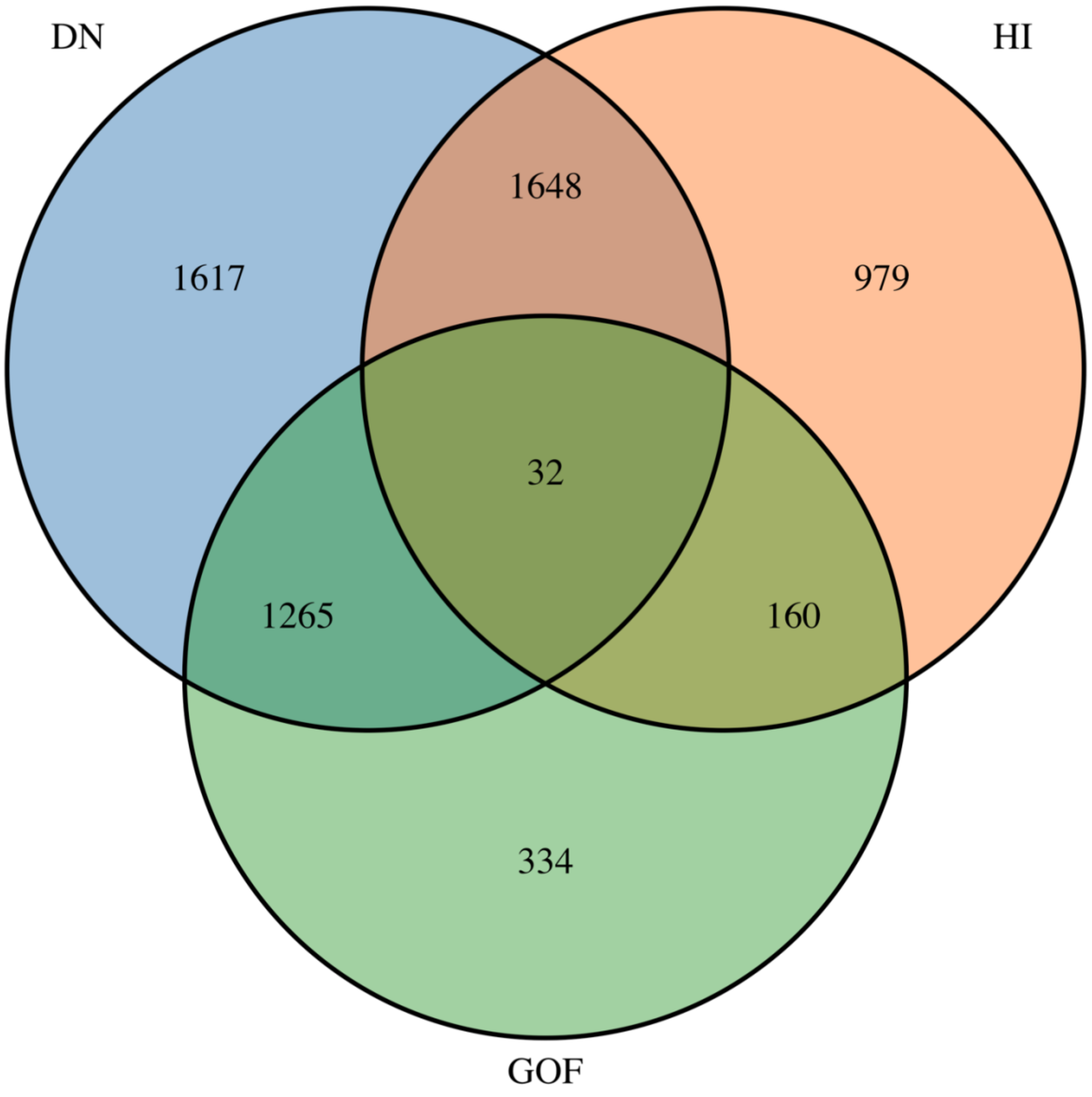

Figure S7: Number of proteins predicted based on the selected GCN model. ADAR proteins were excluded in for this calculation.

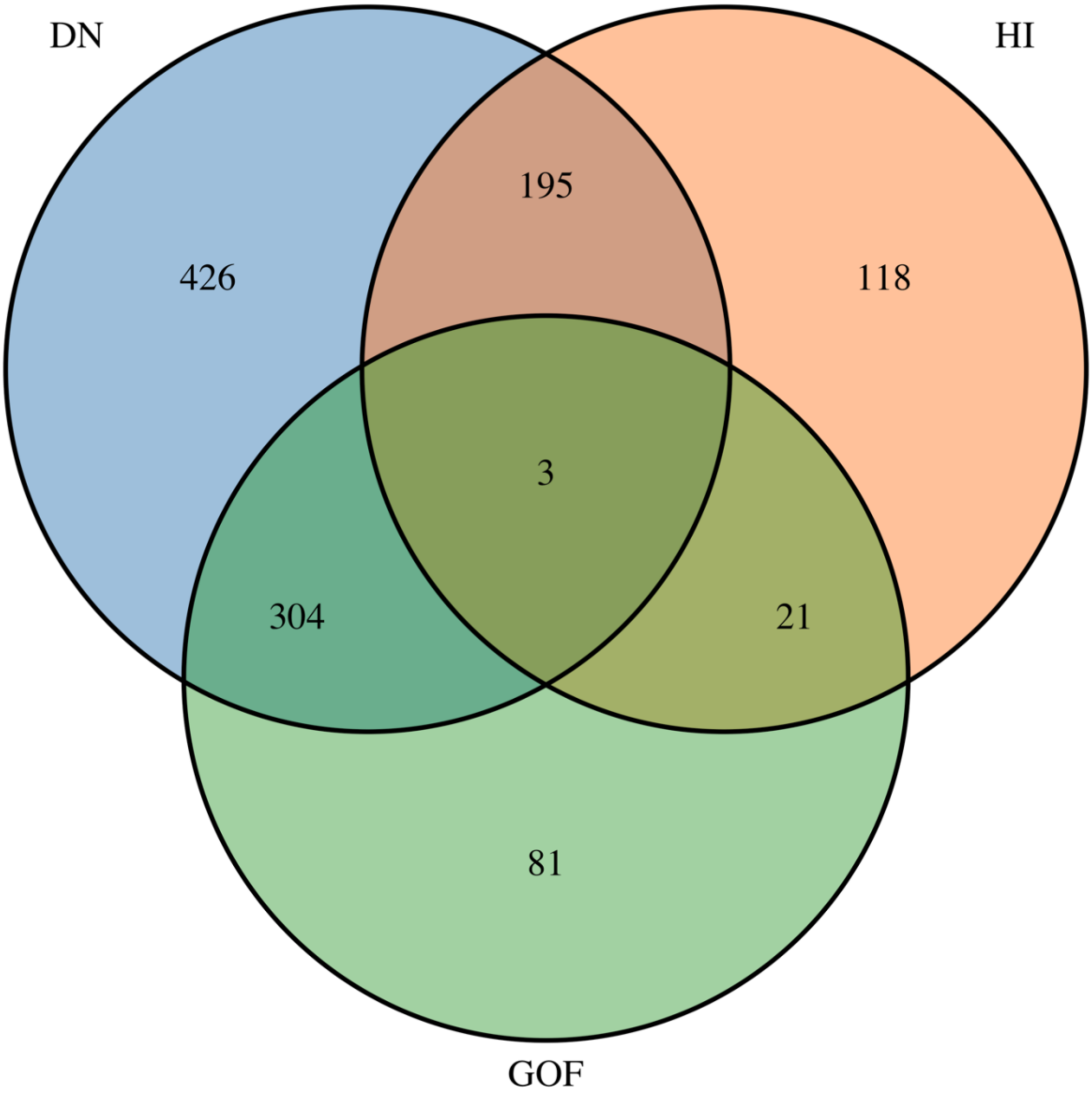

Figure S8: Number of proteins predicted based on the selected GCN model. Only ADAR proteins were included in for this calculation.
